# Adapted Correlation Methods for Laser Speckle Imaging of Microbial Activity: Evaluation and Rationale

**DOI:** 10.3390/s25185772

**Published:** 2025-09-16

**Authors:** Ilya Balmages, Katrina Smite, Dmitrijs Bļizņuks, Aigars Reinis, Alexey Lihachev, Ilze Lihacova

**Affiliations:** 1 Institute of Applied Computer Systems, Riga Technical University, LV-1048 Riga, Latvia; katrina.smite@rtu.lv (K.S.); dmitrijs.bliznuks@rtu.lv (D.B.); 2 Pauls Stradins Clinical University Hospital, LV-1002 Riga, Latvia; aigars.reinis@stradini.lv; 3 Department of Biology and Microbiology, Riga Stradins University, LV-1007 Riga, Latvia; 4 Institute of Atomic Physics and Spectroscopy, Faculty of Science and Technology, University of Latvia, LV-1004 Riga, Latvia; aleksejs.lihacovs@lu.lv (A.L.); ilze.lihacova@lu.lv (I.L.)

**Keywords:** correlation analysis, image processing, laser speckle imaging, microorganism spatiotemporal activity estimation, signal processing

## Abstract

The laser speckle technique provides a non-invasive remote sensing method for monitoring biological dynamics. In this study, we focus on assessing microbial growth through systematic comparison of correlation-based speckle image analysis methods. We compare conventional techniques, NCC, ZNCC, the Lewis method, and Phase correlation, with two newly proposed variants: frequency-domain correlation of normalized images and ZNCC with limited shifts around the peak. We analyze these methods in terms of precision and computational efficiency. Our results demonstrate that the proposed techniques offer optimal trade-offs for tracking subtle microbial activity, particularly in early-stage growth. This paper aims not only to identify the most effective tools for laser speckle analysis, but also to justify the use of laser speckle imaging for microbial activity assessment.

## 1. Introduction

Monitoring microbial activity, especially during the early growth phase, is crucial in both biological research and clinical diagnostics. Rapidly detecting microbial growth is key for determining antibiotic efficacy, infection control, and food safety assessments, yet conventional techniques often rely on endpoint measurements or require invasive sampling, which can delay interventions or miss subtle early changes.

Conventional methods for monitoring microbial activity include colony-forming unit (CFU) assays and spotting assays on agar plates [1], optical density (OD) measurements using spectrophotometry [2], adenosine triphosphate (ATP) bioluminescence assays [3], and microscopic staining techniques [4]. While these approaches are widely established, they suffer from important drawbacks: CFU counts are time-consuming (often requiring 24–48 h), OD lacks sensitivity at low cell densities, ATP-based assays may not distinguish between live and dead cells, and microscopy is labor-intensive. These limitations highlight the need for label-free, real-time techniques that can detect subtle microbial dynamics at an early stage.

The laser speckle technique is a powerful non-contact tool for analyzing dynamic changes by detecting fluctuations in speckle patterns generated by coherent light scattering. Contrast analysis, based on the spatial and temporal statistics of speckle patterns, estimates motion or change through intensity fluctuations that blur the image and reduce contrast [5]. This contrast is directly related to normalized temporal autocorrelation functions [6,7]. Many studies on detecting microbial behavior are based on speckle contrast analysis. In [8], different bacterial types in liquid media were distinguished by analyzing the temporal evolution of the spatial contrast curve. Notably, Ref. [9] combined temporal contrast analysis with correlation, though only as a supporting tool: a mathematical model was proposed that incorporates pixel-to-pixel correlation between consecutive frames to improve measurement accuracy for slow dynamics such as bacterial growth. Study [10] introduced an approach based on a spatiotemporal Fourier transform, where dynamic and static speckle components are separated using 3D low- and high-pass filtering. However, this method is limited by a trade-off between window size and resolution, andand requires adjusting the filtering threshold for each experiment.

While contrast-based methods are effective, correlation analysis provides greater precision in detecting displacements, allowing more sensitive tracking of dynamic events [11], which is particularly important for capturing early microbial dynamics.

In this study, we focus on developing an accurate and computationally efficient approach for detecting microbial activity using correlation-based methods. Unlike the commonly used decorrelation assessment [12,13,14] or approaches based on temporal changes in the correlation coefficient, such as biospeckle activity (BA) [15], our method relies on displacement estimation [16] to enhance sensitivity. We compare four established techniques—Normalized Cross-Correlation (NCC), Zero-Mean NCC (ZNCC), the Lewis method, and Phase Correlation—with two newly proposed alternatives: (1) frequency-domain correlation of normalized images and (2) ZNCC with a limited number of shifts near the peak. The methods are evaluated in terms of accuracy and processing speed, using ZNCC as a reference. The goal is to ensure suitability for large-scale speckle data analysis over extended microbial growth periods. Our approach enables the implementation of a subpixel-resolution correlation method that transforms speckle image sequences into three-dimensional signal arrays [17,18]. This transformation facilitates time-frequency analysis of microbial behavior, offering both sensitivity and interpretability. Study [19] demonstrates how variations in sampling rate affect the values of dynamic speckle indexes and emphasizes the importance of knowing the frequency band of the analyzed phenomenon when choosing the appropriate sampling rate. The authors recommend using the lowest possible rate without compromising speckle resolution. This is a valuable solution, but it relies on indirect and non-intuitive coefficient calculations with various filters. In contrast, our approach converts speckle image sequences into signal arrays, allowing the frequency content to be directly observed (using FFT or spectrogram) and the minimum feasible sampling rate to be determined. Our previous work [20] provides a more detailed analysis of sampling rates for different microorganism types.

To our knowledge, existing studies on microbial activity assessment using the laser speckle technique have not implemented this image-to-signal transformation framework [21,22,23,24,25,26,27]. By introducing accumulated correlation shifts, our method enables temporal and frequency-domain analysis across spatial locations, providing a novel perspective on microbial dynamics, with the potential to improve early detection and monitoring in biological and clinical settings.

## 2. Materials and Methods

### 2.1. The Experimental Setup

The experimental setup included a 10 Mpix CMOS camera (“uEye UI-1492LE-C”, IDS Imaging Development Systems, GmbH, Obersulm, Germany) equipped with a “JHF16M-MP2” lens (Space, Inc., Otawara, Japan). The exposure time was set to one second. Images were captured at 20 s intervals for bacterial experiments and at 1 s intervals for fungal experiments, corresponding to sampling frequencies of 50 mHz and 1000 mHz, respectively. The rationale and detailed calculations for selecting the sampling frequency of microorganism signals are provided in [20]. To generate laser speckles, an expanded 658 nm laser beam was used, ensuring uniform illumination across the entire Petri dish. The light source was a laser diode (“LP660-SF60”, Thorlabs, Inc., Newton, NJ, USA) producing a laser spot with a diameter of 12 cm.

### 2.2. Microbial and Fungal Strains and Cultivation Conditions

Clinical isolates of *Candida albicans* were used in the study and incubated at 37 °C. Fungal inoculation on the medium was performed in accordance with the EUCAST standard procedure [28].

Clinical isolates of *Escherichia coli* (*E. coli*) and *Klebsiella aerogenes (K. aerogenes)* bacteria were selected for a bacterial experiment. To minimize artifacts, all experiments were conducted in a separate room using an incubator maintained at 37 °C. Bacterial suspensions were prepared in saline to a density equivalent to the 0.5 McFarland turbidity standard. Ciprofloxacin disks (CIP, 5 μg) were selected for testing against *E. coli*, and cefotaxime disk (CTX, 5 μg) and ampicillin disk (AM, 10 μg) for *K. aerogenes* were also evaluated experimentally. In accordance with EUCAST guidelines, the bacteria were cultured on Mueller–Hinton agar in Petri dishes [29].

To demonstrate changes within bacterial colonies, *Staphylococcus aureus* ATCC^®^ 6538P, *Escherichia coli* ATCC^®^ 8739 (*E. coli*), and *Vibrio natriegens DSM 759 (V. natriegens)* were selected for this study. Bacterial cultures were maintained on agar plates. *S. aureus* and *E. coli* were maintained on LB agar media consisting of Bacto Peptone 10 g/L, yeast extract 5 g/L, agar 20 g/L (all Biolife, Milan, Italy), and NaCl 5 g/L (Sigma, Taufkirchen, Germany). *V. natriegens* was maintained at room temperature on agarized NB salt broth: Difco Nutrient Broth 8 g/L, NaCl 15 g/L, as suggested by [30], and subcultured from single colonies in liquid media, incubated overnight at 30 °C. Petri dishes were inoculated with serial dilutions to yield 20–200 colonies per plate and then incubated at 30 °C (Herracell 240-i, Thermo Scientific, Langenselbold, Germany).

## 3. Review of Correlation Algorithms for Laser Speckle Analysis

### 3.1. Speckle Displacement Measurement

Digital speckle correlation techniques were first introduced in the early 1980s for analyzing mechanical deformation [31,32]. These methods enable tracking of small deformations, even in the presence of large, rigid-body motion. A reference speckle image is compared to a deformed image, with both divided into small sub-regions. Reliable displacement estimation requires high-contrast and high-frequency random patterns-properties characteristic of laser speckle. The sub-image size determines spatial resolution: larger sizes reduce resolution, whereas small sizes hinder correlation accuracy.

Displacement is estimated for each sub-region using a cross-correlation algorithm (Equation (1)).

(1) $$CC\left(u,v\right)=\;\sum_{x}\sum_{y}\left(\left(a\left(x,y\right)\right)\cdot (b(x-u,y-v))\right)$$

where a(x,y) and b(x,y) are two frames (before and after deformation), u and v are spatial displacements between the two frames in the directions of x and y, respectively.

The accuracy of correlation decreases with increased speckle size. Subpixel precision can be improved by interpolating around the correlation peak, typically using parabolic, Gaussian, or Fourier series expansion methods [33,34].

**Figure 1:**
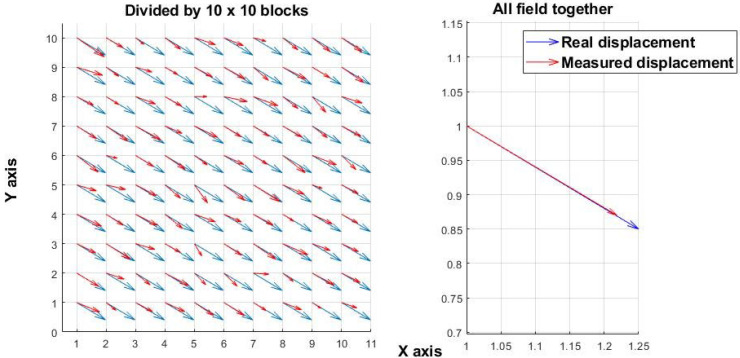
Simulated mechanical displacement. (**Left**): displacement field derived from a speckle image (100 × 100 pixels) subdivided into 10 × 10 blocks. (**Right**): analysis applied to the entire image without subdivision.

**Figure 2:**
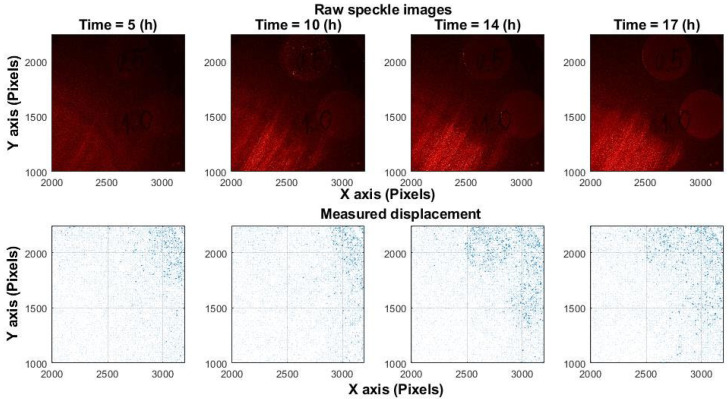
Experimental speckle displacement in *Candida albicans.* (**Top row**): raw speckle image captured at 5, 10, 14, 17 h post-inoculation. Spots with different fungal concentrations observed. Contrast represents intensity changes. (**Bottom row**): displacement field between two frames (1 s interval), subdivided into 10 × 10-pixel blocks.

Figure 1 shows a simulated displacement field resulting from mechanical motion. A 100 × 100-pixel random speckle image was simulated and uniformly shifted along both the x- and y-axes by the same number of pixels, generating two images (before and after the shift). The “real displacement” denotes the applied shift, whereas the “measured displacement” corresponds to the value calculated by the algorithm. In contrast, Figure 2 presents experimental results from *Candida albicans* fungal growth, where displacements are unpredictable and oriented in random directions. Similar displacement patterns have been observed during bacterial growth, confirming that microbial activity induces non-uniform and random motion. Figure 1 and Figure 2 illustrate that analysis performed at only two time points (before and after a change) is effective for assessing mechanical shifts but insufficient for measuring the chaotic processes associated with microorganism activity. The experiments in this study were carried out at a spatial scale of 10–15 µm per pixel.

Frequency-domain cross-correlation methods have also been proposed [34,35], including normalization strategies. Notably, the correlation peak width is approximately twice the speckle size in the image [36], which may introduce errors in displacement estimation.

### 3.2. Signal Reconstruction by ZNCC

Inspired by mechanical deformation detection techniques, a method was developed to detect acoustic vibrations and reconstruct audio signals. Comparative analysis of six algorithms demonstrated that cross-correlation performs particularly well for speech signal reconstruction from speckle patterns [37].

Signal-induced speckle changes can be decomposed into three primary motion components: transverse, axial, and tilt. Under normal imaging conditions with small vibrations, transversal motion is strongly demagnified, producing barely detectable image shifts. Axial motion leaves the speckle pattern essentially unchanged. Thus, the third type of motion that remains is tilt [38]. In controlled conditions, speckle changes reflect displacement without shape deformation, but in noisy environments, this assumption is unreliable.

Displacement is estimated by selecting a template from the first frame and locating its best match in subsequent frames using ZNCC (Equation (2)).

(2) $$ZNCC\left(u,v\right)=\;\frac{\sum_{x}\sum_{y}\left((a(x,y)-\bar{a})*(b(x-u,y-v)-\bar{b})\right)}{\sqrt{\sum_{x}\sum_{y}(a(x,y)-\bar{a}{)}^{2}*\sum_{x}\sum_{y}(b(x-u,y-v)-\bar{b}{)}^{2}}}$$

where a(x,y) is the first (template) frame and b(x,y) represent subsequent frames in the sequence, $\bar{a}$ and $\bar{b}$ are the mean intensity values of the respective regions.

Changes that occur between successive frames are characterized by a displacement in the location of the maximum correlation value (Equation (3)).

(3) u^,v^=argmaxu,vZNCC(u,v)

**Figure 3:**
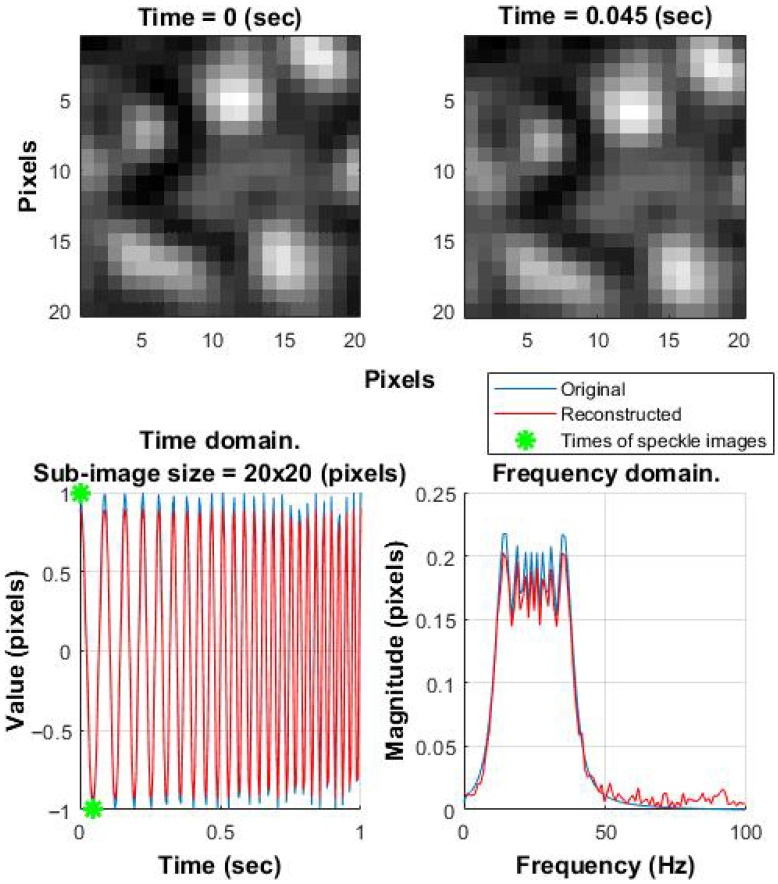
Reconstructed acoustic signal from speckle images. Linear sweep: 10–40 Hz over 1 s, sampling frequency: 200 Hz. (**Left Bottom**): time domain. (**Right Bottom**): frequency domain. The **Left Top** image shows the speckle pattern at time 0 s (marked by the first green asterisk in the **Left Bottom** plot), while the **Right Top** image shows the speckle pattern at time 0.045 s (marked by the second green asterisk in the **Left Bottom** plot).

Subpixel accuracy is achieved by interpolating around the correlation peak, and this process is repeated throughout the speckle image sequence [39,40]. Figure 3 shows a simulation of signal reconstruction from speckle images using a linear frequency sweep (10–40 Hz over 1 s, sampled at 200 Hz). The template is extracted from the first frame, and all displacements are computed relative to this reference. The FFT shows the average energy over the full signal, not instantaneous frequencies. Since the sweep spends little time at the edges (10 and 40 Hz), those frequencies contribute less energy, so the spectrum appears narrower.

The “original” signal is an artificially generated reference used to apply a shift to the constant speckle image. The “reconstructed” signal is obtained by the algorithm. Both signals are shown in the time and frequency domains.

Despite the biological focus of the study, this example demonstrates the potential of using the correlation algorithm for extracting vibration-induced signals, which, after further adaptations, could be applied to signals from microbial colonies.

Although this method enables the analysis of more complex dynamics than simple mechanical shifts, several factors must be considered: the chaotic nature of microbial growth prevents the use of a fixed initial reference frame, and slow spatial expansion eliminates the need for wide-area scanning.

### 3.3. Temporal Decorrelation Effect

While spatial correlation methods detect displacement between frames, temporal and spatiotemporal decorrelation techniques provide insight into dynamic activity. Laser speckle decorrelation, defined as the loss of similarity between speckle patterns over time, is widely used in microbiology and medicine to assess motion within living systems. Living matter leads to spatial and temporal changes, producing dynamics in speckle patterns that can be quantified by analyzing the decay of inter-frame correlation.

The literature describes two approaches for determining decorrelation times:

(1) Pixel-wise autocorrelation, where the normalized temporal intensity correlation for each pixel is calculated over a sequence of frames (Equation (4)) [41].
  (4) $$C(n,k)=\frac{1}{K-k}\sum_{i=1}^{K-k}\frac{I_{n}(t_{i})I_{n}(t_{i}+k)}{\bar{{\bar{I}}_{n}^{2}}}$$
  where K number of images in the sequence, k is the lag time, $I_{n}\left(t_{i}\right)$ is the intensity of pixel n at time $t_{i}$ and ${\bar{I}}_{n}$ represents the temporal averaged intensity of pixel n.

For improved robustness, decorrelation is often calculated over averaged regions rather than individual pixels, as shown in Equation (5) [41].

(5) $$C(\tau )=\frac{1}{N}\sum_{n=1}^{N}C(n,k)$$

This allows tracking local dynamic changes but is sensitive to noise.

(2) An adjusted formulation has also been proposed to account for limited sample size effects [42] (Equation (6)).
  (6) $$C_{t}(k*\delta t,x,y)=\;\frac{\langle I(t,x,y)I(t+k*\delta t)\rangle -\langle I(t,x,y)\rangle \langle I(t+k*\delta t)\rangle }{\sqrt{\left[\langle I^{2}(t,x,y)\rangle -{\langle I(t,x,y)\rangle }^{2}\right]\left[\langle I^{2}(t+k*\delta t,x,y)\rangle -{\langle I(t+k*\delta t,x,y)\rangle }^{2}\right]}}$$
  where x and y are the pixel positions in the image, k is the frame number, δt is the time interval between two adjacent frames.

Typically, the first frame serves as a reference and is compared with subsequent images to assess decorrelation [43,44]. The resulting decorrelation curve characterizes the sample’s activity: living systems exhibit faster decorrelation due to inherent motion. This method has been applied in various contexts, including fruit quality assessment [44], analysis of atherosclerotic plaque elasticity [45], and studies of bacterial behavior [46].

Microbial activity is assessed by analyzing the temporal decay of the correlation peak. Samples containing living microorganisms show rapid decorrelation due to spontaneous motion, while samples without them show minimal change. Study [46] showed that UV radiation, due to its antibacterial properties [47,48], can effectively sterilize samples with high bacterial counts. Prolonged exposure increases correlation values, indicating reduced microbial activity.

**Figure 4:**
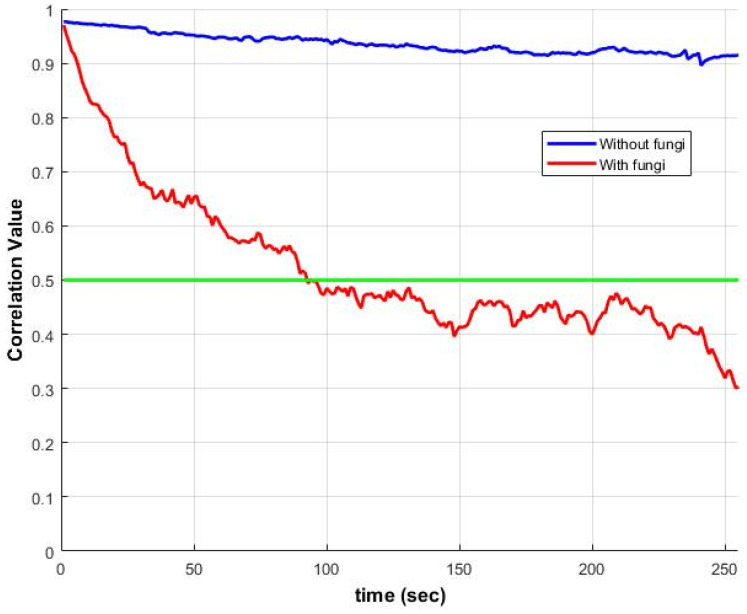
Temporal evolution of ZNCC between the first frame and subsequent frames for two conditions: (1) without fungi (blue) and (2) with *Candida albicans* fungi (red). The green line marks the correlation level of 0.5.

However, the method is sensitive to external vibrations, system instability and evaporation, all of which can also cause decorrelation. Figure 4 illustrates ZNCC changes over time for samples with and without *Candida albicans* fungi. The correlation with the first frame drops below 50% within minutes, underscoring the need to adapt the existing algorithm for analyzing microbial activity.

### 3.4. Summary and Rationale for Method Adaptation

The non-normalized correlation method, originally designed for analyzing uniform mechanical displacements, shows significant limitations when applied to living microorganisms. Microbial activity induces rapid decorrelation, making the fixed first-frame reference approach—commonly used in acoustic signal reconstruction—less effective. Consequently, an alternative correlation strategy is required—one that accounts for decorrelation dynamics, spatial variability, slow spatial expansion, and subpixel sensitivity. The following section introduces an adapted correlation algorithm designed to capture microbial activity as a spatiotemporal signal.

## 4. Converting Laser Speckle Image Sequences into Temporal Signals

Based on earlier sections, we adopt a modified version of the signal reconstruction approach (Section 3.2) to analyze microbial spatiotemporal activity. The goal is to convert sequences of laser speckle images into temporal signals, analogous to the output of a micro-vibration sensor.

Unlike acoustic signal reconstruction, which assumes that speckle patterns shift but retain their shape, microbial activity introduces significant variability. Growth-induced micro-vibrations are local, multidirectional, and cause rapid speckle changes and decorrelation, as shown in Figure 2 and Figure 4. Consequently, using the first image as a fixed reference becomes ineffective. Instead, we compute ZNCC between each pair of consecutive frames (1↔2, 2↔3, …), treating a(x,y) and b(x,y) in Equation (2) as adjacent frames.

Since correlation is performed between successive image pairs rather than against a fixed template, the calculated displacements are accumulated along the x and y axes to enable accurate signal reconstruction (Equation (7)).

(7) sign=∑i=1nδ^i

where δ^ are all previous displacements.

Unlike acoustic signals, microbial displacements are low in amplitude, which allows correlation to be computed at fixed locations without spatial scanning. The field is divided into N × N sections, and ZNCC with subpixel interpolation is applied to each [49].

**Figure 5:**
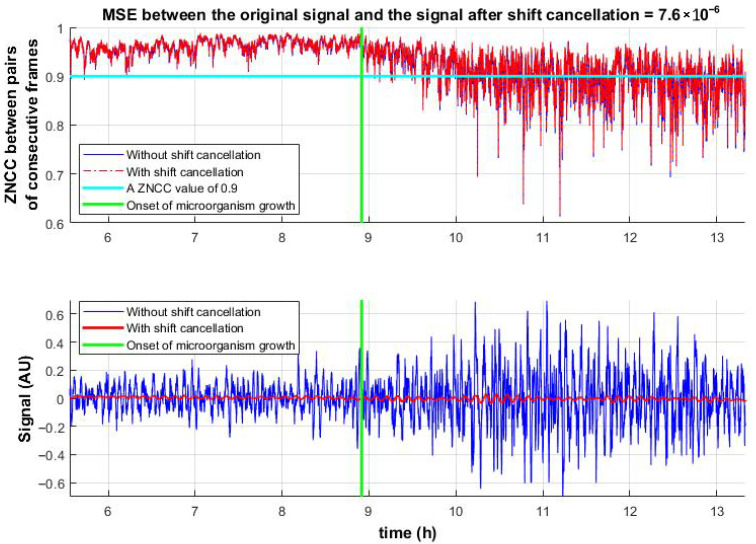
ZNCC peak values between successive frame pairs before and after shift cancelation (**top**). Corresponding temporal signals derived from peak displacements (**bottom**) before and after shift cancelation.

Figure 5(top) shows the temporal evolution of ZNCC values between consecutive frame pairs, clearly distinguishing between the presence and absence of microbial activity. This highlights the advantage of the proposed method over the decorrelation-based approach (Figure 4).

Figure 5(bottom) presents the reconstructed activity signals. Since microbial growth occurs directly on the observed surface, in addition to micro-vibrations, rapid changes in the speckle pattern are observed. To assess the influence of these structural changes, shift compensation (inverse image shifting, Equation (8)) was applied.

(8) $$f_{shifted}(x,y)={FT}^{-1}\{IMG(u,v)\cdot e^{-j2\pi (\frac{u∆x}{N}+\frac{v∆y}{M})}\}$$

where IMG(u,v) is the 2D Fourier transform of the original 2D image; ${FT}^{-1}$—inverse Fourier transform; N, M are the width and height of the image; u, v are the frequency indices in the FFT; Δx, Δy are the subpixel shifts along the x and y axes.

The shift-canceled ZNCC values were compared to those before compensation. The results confirm that micro-vibrations have minimal impact on ZNCC peak values, indicating that the correlation drop is primarily due to pattern changes. At the same time, micro-vibrations induced by microbial activity affect the peak location, enabling the identification of active microbial regions.

**Figure 6:**
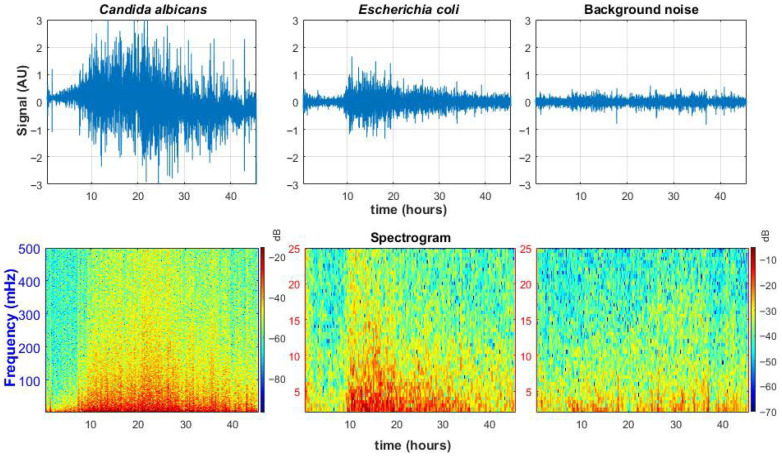
Temporal signals reconstructed from speckle images. Top row: signals from a *Candida albicans* colony (**left**), *E. coli* colony (**center**), and background (**right**). Bottom row: corresponding spectrograms. Time window length = 0.5556 (h), window function is Hamming, overlap = 90%, achieved frequency resolution = 0.5 (mHz).

Figure 6 compares reconstructed signals inside bacterial and fungal colonies as well as outside. Spectrogram analysis reveals distinct activity signatures, confirming that microbial growth generates very low-frequency signals.

Figure 6 highlights two key differences between fungal and bacterial activity:

1. Fungal signals show greater amplitude, suggesting more pronounced structural changes over time. This likely reflects the larger size and multicellular organization of fungi, including hyphal growth and branching, which induce stronger fluctuations in the speckle pattern.
2. Compared to bacteria, which grow primarily through binary fission and exhibit slower, more uniform expansion with lower-amplitude and lower-frequency signal profiles, fungi tend to produce richer and higher-frequency signals due to their more complex morphology, diverse developmental stages, and dynamic growth behaviors such as hyphal branching, budding, and sporulation [50].

This section introduces algorithmic modifications specifically tailored to displacement analysis for detecting and distinguishing microbial activity. The subsequent section evaluates the accuracy and computational efficiency of both conventional and proposed correlation methods.

## 5. Measurement Accuracy and Processing Time of Correlation Algorithms

This section evaluates various implementations of cross-correlation algorithms with respect to accuracy and computational efficiency. Tests were conducted on 10 × 10 pixel image sections, representing 100–150 µm × 100–150 µm of physical area.

### 5.1. Normalization and Correlation Approaches

When image brightness fluctuates due to lighting or exposure, normalization becomes essential. It enhances the robustness of correlation methods by reducing sensitivity to amplitude and offset variations [51], whereas non-normalized correlation, which, while faster, is more sensitive to such variations.

We compare six implementations:

(1) NCC (Equation (9)): NCC can be implemented by computing non-NCC (Equation (1)) first and then applying normalization, which scales the result to the −1 to 1 range while retaining non-NCC’s sensitive to amplitude variations.
  (9) $$NCC(u,v)=\;\frac{\sum_{x}\sum_{y}\left((a(x,y))*(b(x-u,y-v))\right)}{\sqrt{\sum_{x}\sum_{y}(a(x,y){)}^{2}*\sum_{x}\sum_{y}(b(x-u,y-v){)}^{2}}}$$
(2) ZNCC (Equation (2)): As described in [52], normalized correlation is commonly represented in two forms: standard NCC and zero-mean NCC (ZNCC) (Equation (2)). It is precisely by subtracting the local mean that ZNCC compensates for brightness variations, making it more robust.
(3) Phase correlation (Equation (10)): Frequency-domain method with magnitude normalization; very fast, but less accurate under deformation [53].
  (10) $$PC(u,v)=FT^{-1}\left(\frac{\left[FT\left[a\left((x,y\right)\right]*\;{FT\left[b\left((x,y\right)\right]}^{*}\right]}{\left|FT\left[a\left((x,y\right)\right]*FT\left[b\left((x,y\right)\right]\right|}\right)$$
  where FT^−1^ and FT—inverse and direct Fourier transform, and ()*—complex conjugate.
(4) The Lewis method [54] performs a fast non-NCC in the frequency domain, followed by a mathematical transformation that converts the non-normalized to a normalized correlation. The method yields accuracy similar to that of standard ZNCC. Fast compared to other methods for large matrices, but less efficient for small matrices.
(5) Proposed method I (Equation (11)): Correlation in the frequency domain using normalized images. Matches ZNCC accuracy, comparable to the speed of phase correlation (which lacks sufficient accuracy), and significantly outperforms the Lewis method in processing time.
  $$An\left(x,y\right)=\frac{a\left(x,y\right)-\bar{a}}{\sqrt{\sum_{x}\sum_{y}(a(x,y)-\bar{a}{)}^{2}}};$$
  $$Bn\left(x,y\right)=\frac{b\left(x,y\right)-\bar{b}}{\sqrt{\sum_{x}\sum_{y}(b(x,y)-\bar{b}{)}^{2}}};$$
  (11) $$ZNCC\left(u,v\right)=FT^{-1}\left(\left[FT\left[An\left((x,y\right)\right]\cdot \;{FT\left[Bn\left((x,y\right)\right]}^{*}\right]\right)$$
(6) Proposed method II: This approach uses the ZNCC formula (Equation (2)) but applies only a limited range of shifts near the image center to locate the correlation peak. For large images, it offers faster performance than the frequency-domain method, making it especially appealing. For smaller images, however, the frequency-domain approach remains slightly faster.

### 5.2. Results and Analysis

#### 5.2.1. Accuracy Analysis

According to the discussion in Section 4, microorganism growth induces two types of changes in speckle image sequences: (1) displacement, which is captured by the proposed correlation methods and (2) random pattern variations, which are not addressed by these methods. To evaluate the influence of pattern changes, displacement was canceled using Equation (8). As shown in Figure 5(bottom), this procedure significantly reduced shifts, leaving only minor residual noise, substantially smaller than the microbial signal.

**Figure 7:**
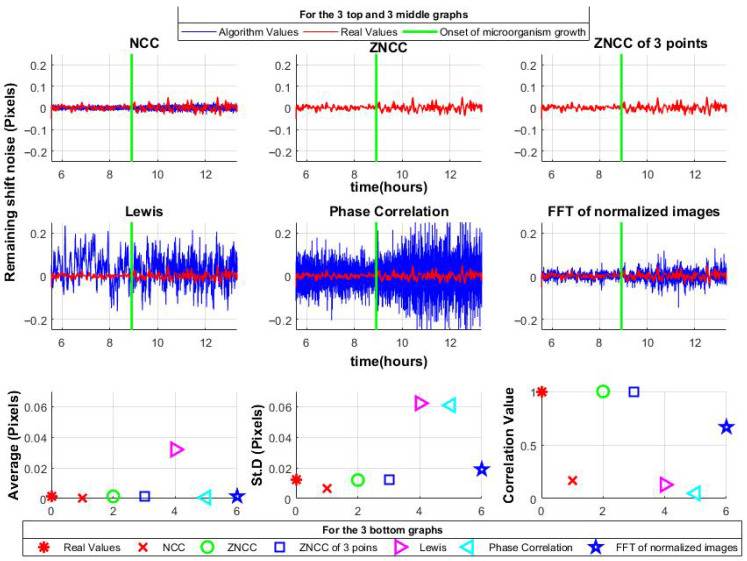
Comparison of various correlation methods applied to speckle images after displacement cancelation. The (**top**,**middle**) rows show results from different algorithms (in the case of ZNCC, the blue and red lines are identical); the (**bottom**) row presents the mean values, standard deviations, and correlation with the original data for each method.

The algorithms were then applied to the shift-canceled data. ZNCC and ZNCC with a limited number of shifts accurately reconstructed the noise. NCC and proposed method I, correlation in the frequency domain using normalized images, showed slight deviations but maintained the mean and standard deviation close to the original. Notably, the proposed method also retained the signal shape, unlike NCC. In contrast, the Lewis method and Phase Correlation exhibited greater variability; moreover, the Lewis method shifted the mean, indicating asymmetry. Results are summarized in Figure 7.

**Figure 8:**
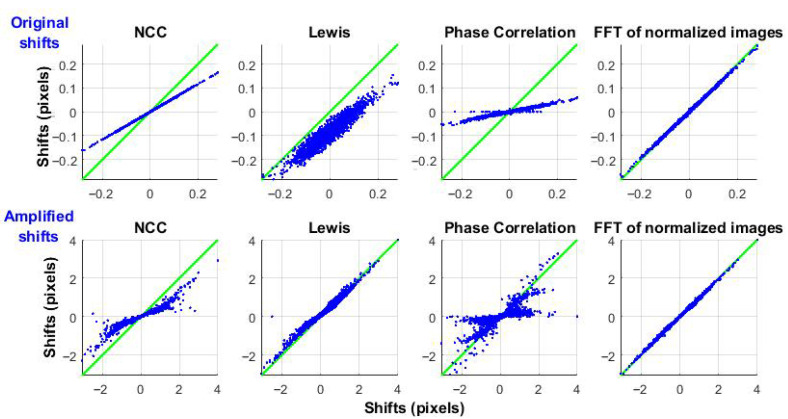
Comparison of different correlation methods applied to a constant speckle pattern shifted by the original displacements (**top row**) and by displacements amplified 10 times (**bottom row**). The green line represents the desired (template) result, while the blue dots indicate the actual values.

It is also important to evaluate how different correlation algorithms respond to microorganism-induced vibrations (displacements) without changes in the underlying speckle pattern. To isolate this effect, a single speckle frame with a constant pattern was synthetically shifted, using Equation (8), based on the previously detected displacements. To clearly demonstrate the results, Figure 8 shows the input-output relationship: the input is the reference displacement obtained using ZNCC, and the output is the response of each algorithm.

The top row shows the original displacements, highlighting noticeable differences in NCC and Phase Correlation compared to the reference. The Lewis method exhibits both high variability and an offset in the mean value.

The bottom row presents displacements amplified by a factor of 10 (comparable to the pixel size). NCC and Phase Correlation show even larger errors, while the Lewis method performs better for larger shifts.

The proposed method I (Frequency-domain correlation using normalized images), demonstrates good accuracy. ZNCC and the proposed method II (ZNCC with limited shifts) serve as reference benchmarks.

#### 5.2.2. Processing Times Analysis

The growth of microorganisms is a slow process that requires imaging large areas (e.g., an entire Petri dish) over many hours. This generates large datasets with numerous frames, leading to long processing times; therefore, computational efficiency is crucial.

It is also worth noting that the proposed method demonstrates high precision even at low SNR, which further confirms its stability and robustness [55]. Consequently, the proposed method holds potential for broader applications in speckle-based detection—particularly in domains where performance time is even more critical (for example, acoustic signal detection (Figure 3)).

Table 1, Table 2 and Table 3 summarize processing times for different correlation algorithms and image sizes. Each value reflects the average time for 5000 processed regions, computed on a standard PC (Intel Core i5-4210U @ 1.70GHz).

The tests were limited to a maximum size of 100 × 100 pixels, as this already demonstrates the essential scaling behavior. Increasing the region size from 10 × 10 to 100 × 100 pixels raises computation time by nearly three orders of magnitude, making larger sizes both computationally impractical and difficult to present in plots, even on a logarithmic scale.

All tests were performed on a single CPU core to keep methods directly comparable. While parallel processing could reduce times, not all algorithms benefit equally, so such comparisons would not be fair or within the scope of this work.

Although Phase Correlation offers fast processing, its inaccuracy under image deformations and displacements makes it unsuitable for this study. NCC also lacks precision. Therefore, the analysis focuses on the remaining four algorithms: ZNCC (used as the reference), the Lewis method, and the two proposed approaches: frequency-domain correlation of normalized images and ZNCC with a limited number of shifts.

Table 1, Table 2 and Table 3 show that while the Lewis method is slower than ZNCC for small matrices (<10 × 10), it outperforms ZNCC on larger ones. Both proposed methods outperform ZNCC and Lewis in speed across all matrix sizes, maintaining accuracy and making them particularly well suited for large-scale or real-time microbial activity analysis.

### 5.3. Practical Applications in Microorganism Research

**Figure 9:**
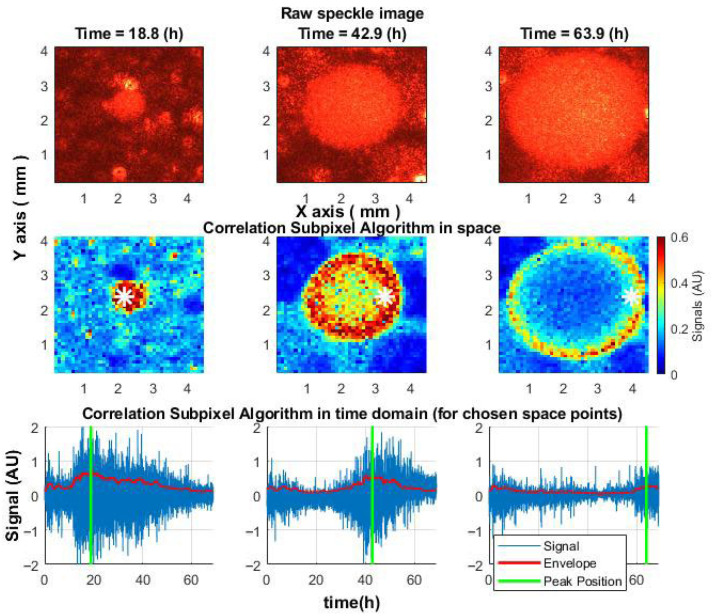
Spatiotemporal dynamics of the speckle signal in an *S. aureus* colony. (**Top**): raw speckle images. (**Middle**): activity changes during growth. (**Bottom**): signal over time at selected points (white star). The green line marks peak activity.

The proposed algorithm, which is based on measuring correlations between displacements in each pair of subsequent frames and accumulating them into signal arrays, revealed that microbial activity within the colonies first peaked at the colony center and then propagated outward, forming a migrating “activity ring”—a feature not visible in raw speckle images. This spatiotemporal pattern was consistently observed in *S. aureus*, *E. coli*, and *V. natriegens* colonies, and mapping the onset of activity decline allowed the reconstruction of colony growth dynamics (Figure 9) [17].

**Figure 10:**
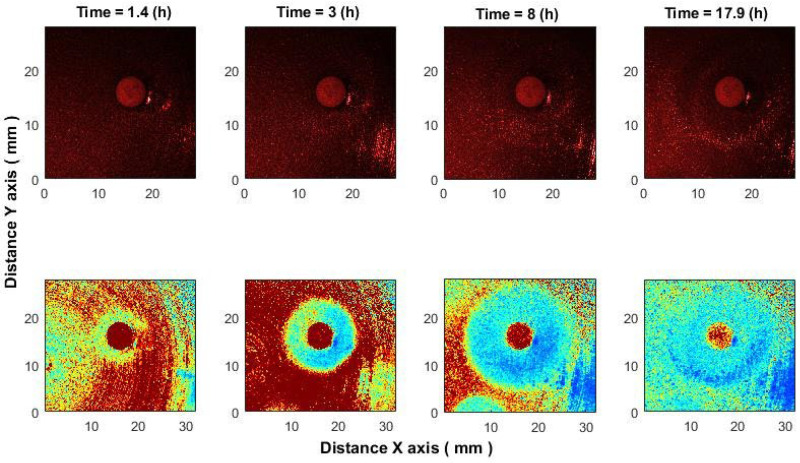
Evolution of the inhibition zone in *E. coli* under CIP (5 μg). (**Top**): raw speckle images. (**Bottom**): after the algorithm applying.

In the antimicrobial susceptibility application, correlation analysis detected inhibition zones within 1–1.5 h after antibiotic placement, significantly earlier than what is visible in raw images. While raw speckle images show only bacterial presence, the algorithm captures activity loss, making inhibition zones clearly visible [18]. In processed images (Figure 10, bottom row), blue indicates low activity and red indicates high activity, with the antibiotic area appearing red due to unrelated chemical processes.

We demonstrate the effect of ciprofloxacin (CIP, 5 μg) on *E. coli*; comparable results were observed with cefotaxime (CTX, 5 μg) and ampicillin (AM, 10 μg) against *K. aerogenes*.

Earlier detection of the inhibition zone can be explained as follows: speckles act as a highly sensitive “ruler,” where even nanometer-scale displacements of scatterers cause measurable pattern changes. The proposed correlation algorithms track frame-to-frame shifts with sub-pixel accuracy (Section 3.1 [33,34]) and remain effective even at low SNR [55].

In practice, the method captures speckle shifts reflecting microbial activity—metabolic micro-motions, morphological changes, or micro-vibrations in the medium. Importantly, it detects the bacterial response to antibiotics rather than the antibiotic itself, observed as reduced activity and the early expansion of the inhibition zone.

## 6. Discussion

This study evaluated multiple correlation-based techniques for analyzing laser speckle images to assess microbial activity. While traditional approaches, originally designed for mechanical systems, assume static speckle patterns and use a fixed reference frame, such assumptions do not hold in biological contexts. Microbial colonies produce non-uniform, multidirectional displacements and dynamically evolving speckle patterns (Figure 1 and Figure 2), leading to rapid temporal decorrelation (Figure 4).

To overcome these limitations, a frame-to-frame correlation approach was proposed, computing displacements between consecutive images and accumulating them to reconstruct continuous temporal signals. This method accounts for both speckle shifts and pattern changes, enabling accurate signal extraction from complex biological systems.

Our comparative analysis showed that phase correlation, although computationally efficient, lacks the precision needed to track biological activity accompanied by pattern changes. Similarly, standard NCC was insufficiently robust against intensity fluctuations. Among the accurate methods, the Lewis algorithm (which shows good accuracy for larger displacements close to pixel size, but lacks precision for small shifts typical of microorganism activity) was surpassed by two proposed alternatives:

- Frequency-domain correlation of normalized images;
- ZNCC with limited shifts around the peak.

Both proposed methods demonstrated high accuracy and fast processing. The Frequency-domain correlation of normalized images was 1.2 times faster for 6 × 6 matrices and 1.15 times faster for 10 × 10, while ZNCC with limited shifts outperformed on larger matrices (1.2 times faster for 100 × 100), with additional gains expected for even larger sizes. These methods enable rapid and precise detection of microorganism activity and behavior, supporting early-stage identification [49].

Biological validation demonstrated that *Candida albicans* colonies generated signals with both higher amplitude and broader frequency content than *E. coli*. This likely reflects the inherently dynamic nature of fungal growth, including hyphal elongation and sporulation [50], as opposed to the relatively uniform binary fission of bacteria.

Speckle patterns reflect microbial growth mechanisms and can visualize specific aspects of microbial activity, such as zones of proliferation within the colony and cellular motility. Fungal hyphae create heterogeneous surfaces that generate speckle patterns with high spatial complexity and slow temporal changes compared to the patterns produced by colonies undergoing rapid bacterial binary fission. Motile bacteria can introduce additional high-frequency variations, further distinguishing bacterial from fungal signatures. These differences suggest that speckle dynamics can serve as non-invasive fingerprints of microbial colony structure and growth, enabling quantitative assessment of specific microbial activities.

These findings confirm the proposed method’s capability not only to detect microbial activity but also to differentiate between microbial types based on their unique temporal and spatial signatures [17]. Our earlier studies demonstrated: (1) a correlation between the proposed method and CFU measurements, enabling early colony detection [49]; (2) quantification of colony radius and internal dynamics [17]; and (3) as shown in this work, characteristic time-dependent signal changes (Figure 9(bottom); Figure 6(top)) that are proportional to microbial growth phases, including lag, exponential, and stationary. However, it should be noted that the current study does not yet directly correlate speckle image or signal features with additional microbial parameters such as optical density, biomass, or hyphal fraction. Establishing these correlations is a critical next step to fully interpret laser speckle signals in terms of biological growth status.

For the successful treatment of patients with severe infections, it is vitally important to determine the most effective antibiotic as quickly as possible. This is especially critical for infections caused by multidrug-resistant bacteria, where the antibiotic resistance profile and potential resistance mechanisms must be determined as rapidly as possible. Methods based on the disk diffusion test are widely used for this purpose. Therefore, integrating this technique into automated antimicrobial susceptibility workflows, particularly those that utilize the disk diffusion test, has the potential to reduce incubation times from 24 h to just 4–5 h [18,56], offering substantial benefits for clinical diagnostics. Moreover, precise management of the antibiotic application time in the bacterial culture allows inhibition to be detected even earlier (Figure 10).

## 7. Conclusions

We present an efficient and accurate methodology for assessing microbial activity using laser speckle correlation. By adapting correlation algorithms to biological dynamics and applying them between consecutive frames, we overcome limitations of fixed reference-frame approaches.

Two proposed methods—frequency-domain correlation of normalized images and ZNCC with limited shifts—demonstrated the best trade-off between accuracy and processing speed, outperforming standard techniques across all tested matrix sizes. The frequency-domain correlation of normalized images is slightly faster for small matrices, while ZNCC with limited shifts is better suited for larger matrices.

The proposed approach enables early detection of microbial growth and differentiation between microbial types based on their unique temporal signal profiles. Fungi exhibit stronger and faster dynamics than bacteria, reflected in higher signal amplitude and frequency content.

In diagnostic microbiology, this technique can accelerate antimicrobial susceptibility testing. When combined with the disk diffusion method, it offers faster, more precise results, improving patient care through timely and targeted therapy.

## Figures and Tables

**Table 1:** Processing time (µs) for 6 × 6-pixel sections.

Method	Average Time (µs)	Standard Deviation (µs)
NCC (Equation (9))	148.8	23.0
Original ZNCC (Equation (2))	168.9	25.1
ZNCC using 3 shifts around the peak	78.8	13.1
Lewis method	478.0	49.1
Phase correlation (Equation (10))	34.5	6.6
Frequency-domain correlation of normalized images (Equation (11))	63.6	11.0

**Table 2:** Processing time (µs) for 10 × 10-pixel sections.

Method	Average Time (µs)	Standard Deviation (µs)
NCC (Equation (9))	515.9	117.3
Original ZNCC (Equation (2))	550.0	150.4
ZNCC using 3 shifts around the peak	93.9	82.7
Lewis method	545.5	78.5
Phase correlation (Equation (10))	48.0	34.7
Frequency-domain correlation of normalized images (Equation (11))	81.8	64.9

**Table 3:** Processing time (ms) for 100 × 100-pixel sections.

Method	Average Time (ms)	Standard Deviation (ms)
NCC (Equation (9))	473.8	30.5
Original ZNCC (Equation (2))	476.8	27.4
ZNCC using 3 shifts around the peak	0.9	0.1
Lewis method	13.0	1.4
Phase correlation (Equation (10))	1.3	1.5
Frequency-domain correlation of normalized images (Equation (11))	1.1	0.2

## Data Availability

The raw data supporting the conclusions of this article will be made available by the author upon request.

## Abbreviations

The following abbreviations and symbols are used in this manuscript:

NCC	Normalized Cross-Correlation
Non-NCC	Non-Normalized Cross-Correlation
UV	Ultraviolet
ZNCC	Zero-mean Normalized Cross-Correlation
PC	Phase Correlation
FT	Fourier Transform
u,v	Spatial displacements between the two frames in the directions of x and y
Δx, Δy	Subpixel shifts along the x and y axes
